# Joint-specific changes in locomotor complexity in the absence of muscle atrophy following incomplete spinal cord injury

**DOI:** 10.1186/1743-0003-10-97

**Published:** 2013-08-15

**Authors:** Brian K Hillen, Gary T Yamaguchi, James J Abbas, Ranu Jung

**Affiliations:** 1 Department of Biomedical Engineering, Florida International University, 10555 W Flagler St, EC 2602, Miami, FL 33174, USA; 2 Center for Adaptive Neural Systems, Arizona State University, 1100 E. University Dr., Ste. 116, Tempe, AZ 85281, USA; 3 Exponent, 23445 North 19th Ave., Phoenix, AZ 85027, USA

**Keywords:** Spinal cord injury, Complexity, Locomotion, Rat, Permutation entropy, Atrophy, Joint kinematics, Gait

## Abstract

**Background:**

Following incomplete spinal cord injury (iSCI), descending drive is impaired, possibly leading to a decrease in the complexity of gait. To test the hypothesis that iSCI impairs gait coordination and decreases locomotor complexity, we collected 3D joint angle kinematics and muscle parameters of rats with a sham or an incomplete spinal cord injury.

**Methods:**

12 adult, female, Long-Evans rats, 6 sham and 6 mild-moderate T8 iSCI, were tested 4 weeks following injury. The Basso Beattie Bresnahan locomotor score was used to verify injury severity. Animals had reflective markers placed on the bony prominences of their limb joints and were filmed in 3D while walking on a treadmill. Joint angles and segment motion were analyzed quantitatively, and complexity of joint angle trajectory and overall gait were calculated using permutation entropy and principal component analysis, respectively. Following treadmill testing, the animals were euthanized and hindlimb muscles removed. Excised muscles were tested for mass, density, fiber length, pennation angle, and relaxed sarcomere length.

**Results:**

Muscle parameters were similar between groups with no evidence of muscle atrophy. The animals showed overextension of the ankle, which was compensated for by a decreased range of motion at the knee. Left-right coordination was altered, leading to left and right knee movements that are entirely out of phase, with one joint moving while the other is stationary. Movement patterns remained symmetric. Permutation entropy measures indicated changes in complexity on a joint specific basis, with the largest changes at the ankle. No significant difference was seen using principal component analysis. Rats were able to achieve stable weight bearing locomotion at reasonable speeds on the treadmill despite these deficiencies.

**Conclusions:**

Decrease in supraspinal control following iSCI causes a loss of complexity of ankle kinematics. This loss can be entirely due to loss of supraspinal control in the absence of muscle atrophy and may be quantified using permutation entropy. Joint-specific differences in kinematic complexity may be attributed to different sources of motor control. This work indicates the importance of the ankle for rehabilitation interventions following spinal cord injury.

## Background

In order to target and quantify the overall pattern of changes in locomotion (particularly the results of decrease in supraspinal control), we assessed 3D locomotor kinematics and kinematic complexity in rats following mild-moderate incomplete thoracic spinal contusion. As a result of spinal cord injury, the connections between the brain and the spinal circuitry below the injury are disrupted. This leads to adaptations in the neurons of the brain and spinal cord as well as changes to the sensory afferents and motoneurons [1-3]. Along with these neural changes, the muscles in the distal limb undergo changes similar to those seen in many disuse paradigms. For example, the muscles tend to atrophy and muscle fibers shift towards faster twitch, more fatigueable ones [4-7]. These effects on muscle properties have been well studied in humans and other animals for a variety of disuse paradigms including weightlessness, bed rest, stroke, partial body support and constrained limbs [8-10]. As spinal cord injury related muscular and neural impairments affect the legs, locomotion is often used as a measure of impairment and recovery.

Both musculoskeletal and nervous system impairments may contribute to the behavioral impairments seen following incomplete SCI (iSCI). While one might expect aberrant neural control to dominate locomotor impairments following iSCI, data in humans has suggested that timing of voluntary initiation of ankle movement (control) is unaltered and loss of muscle strength (specifically dynamic muscle strength) may be responsible for maladaptive changes in ankle gait patterns [11,12]. However, dynamic muscle strength (for instance, time to maximal contraction) may rely on neural control, not solely muscle strength. Additionally, neural control of muscle in an isolated, voluntary, movement is significantly different than movement during gait [13].

Following stroke, the overarching control system for gait is simplified. Even in normal individuals, synergies in muscle activations during locomotion are common [14]. For instance, knee extensors and hip abductors activate at the same time to provide body support during stance [15]. While these synergies exist in normal subjects, they are numerous and varied. Following stroke, these synergies collapse to just one pair: one for flexion and one for extension of the whole limb, indicating a decrease in motor control complexity [16]. This decrease in complexity may be due to unmasking of the primitive gait controller in the spinal cord when there is reduced input from supraspinal centers [17]. This idea is one of some debate however, with an alternative hypothesis that these synergies are purely due to constraints in the task being performed [18].

As iSCI also results in disruption of supraspinal motor control of the limbs, a similar muscle synergy and loss of complexity effect could be seen following injury. There is evidence that much of the coordination associated with locomotion resides in the spinal cord from studies in deafferented and decerebrate preparations [19], but no current study has assessed this effect in SCI subjects during locomotion. Existing reviews on the subject of motor synergy following spinal cord injury also use the stroke framework [20] due to the sparsity of information regarding spainal cord injury. Initial reports on muscle synergies following spinal cord injury have assessed hand movements for activities of daily living and, while differences are noted, change in quantity has not been reported [21].

There are a number of measures that can be used to analyze time series data such as joint angle locomotor data for synergy and synchronization. Synchronization of cyclic joint movement can be measured with principal components analysis (PCA) and alterations in phase delay between the joint motions. PCA assesses the quantity of information in the signal and how similar time series are to one another [22]. As the joints (hip, knee, and ankle) become synchronized, fewer and fewer components would be required to describe the motion of all three joints. Joint synchronization can also be measured by examining the phase delays between the activation of joints within a limb. As the phase delay becomes closer to 0 (exactly in phase), the joint motions become more synchronized. Kinematic complexity can be measured on a joint by joint basis by looking at permutation entropy of the joint angle time series. This measure assesses the quantity of the information in the signal by identifying how likely it is for the joint angle to continue in the same direction from one time point to the next [10].

The rat model is currently one of the most studied and best understood models of SCI being used [23,24]. Many researchers have studied the effects of SCI in the rat on a number of functional impairments and measured the effects of injury at multiple levels of physiology [1] and behavior [25]. The general time course of rat hindlimb locomotor recovery following SCI has been characterized using the Basso, Beattie, Bresnahan locomotor recovery score [26]. However, there has been relatively little research into specific hindlimb kinematics, with some notable exceptions [27-30]. Also, little information has been collected on the muscles in the rat hindlimb, usually only a few muscles per study [4,31,32].

This study addresses the hypothesis that iSCI impairs gait coordination and decreases locomotor complexity at each hindlimb joint and the entire hindlimb. This is accomplished by assessing complexity of 3D joint kinematics during treadmill locomotion and measuring muscle parameters in rats four weeks following either a sham injury or a mild-moderate spinal cord contusion injury. To our knowledge, no previous study has analyzed changes in kinematics complexity along with known levels of muscle atrophy. The analyzed data indicate that spinal contusion injury leads to impaired coordination and decreases in locomotor complexity on a joint-specific basis. The absence of changes in muscle parameters indicates that these locomotor changes can be due purely to losses in supraspinal drive.

## Methods

Experimental data were collected from 12 female, adult, Long-Evans rats (270–300 g). Six rats received sham injuries and six received a mild-moderate spinal cord contusion injury. All rats were kept on a 12 hour light/dark cycle with ad libitum food and water. The study was approved by the Institutional Animal Care and Use committee (IACUC) of Arizona State University and complies with the Guide for the Care and Use of Laboratory animals.

### Spinal cord injury surgery

Rats were randomly selected to undergo T8 vertebral (T9 spinal) sham or mild-moderate incomplete contusion injury (iSCI). Surgery was performed under aseptic conditions similar to Scheff et al. [33]. Rats were anesthetized under 1-2% isoflourane and given an injection of the analgesic buprenorphine (0.02–0.05 mg/kg). The vertebral process of T8 was removed and a U-shaped laminectomy was performed. For animals in the contusion (iSCI) group, T7 and T9 were clamped in place and a mild-moderate contusion (154 ± 3 kdynes SD) was performed with an IH Instruments force controlled impactor for a T9 spinal level contusion. Following contusion or laminectomy, the muscles were closed in layers using resorbable sutures and the skin closed with wound clips (which were removed 1–2 weeks following surgery). The animal was then given injections of 5 cc of sterile saline, and 33.3 mg/kg of Cefazolin antibiotic and allowed to wake slowly on a heated pad.

Animal care post-surgery consisted of twice daily bladder expression until the animal was able to void on its own along with twice daily injections of saline, buprenorphine (as above) and antibiotic (as above) for the first 7 days following injury. Saline administration was continued if the animal remained dehydrated and antibiotic administration continued an additional week if the animals showed signs of urinary tract infection from a urinalysis test strip. Following surgery, animals were allowed to move freely in their cages for 4 weeks. Behavioral analysis consisting of the Basso, Beattie, and Bresnahan (BBB) 21 point locomotor score [26] was collected on the animals every day for the first week and each week thereafter in order to verify injury severity. Rats were observed for 4 min by two experimenters in order to score hindlimb function. The scale takes into account milestones representative of locomotor recovery including motion in the leg, paw position, coordination and weight support. Experimenters were not blinded to the group of the animal as the differences were clearly evident to trained individuals.

### Hindlimb treadmill kinematics

Prior to the spinal cord surgery, animals were allowed to acclimatize to their new environment for one week and trained to walk on a treadmill (Columbus Instruments) for 2 days for 10 min each at a progression of speeds from 0–21 meters/min. 3D kinematic data was collected 4 weeks post injury on both sets of animals as described previously [27]. Briefly, animals were anesthetized under 1-2% isoflourane, shaved as necessary, and cone-shaped reflective markers were attached bilaterally to the bony prominences of the ankle (lateral malleolus), knee (femoral condoyle), hip (greater trochanter and iliac crest), shoulder (greater tubercle), elbow (lateral epicondyle), and wrist (ulnar head). In addition, a strip of reflective tape was placed around the fifth metatarsal of the hindlimb, close to its attachment. The markers were not spherical, as is desirable for centroid calculations, but their small size (approximately 5 mm) minimized any error. Markers were placed while the animal lay on its side. Video was recorded from 4 infrared-sensitive cameras with co-localized infrared light sources (2 on each side of the animal) at 60 Hz. A 36 point static calibration object was first recorded. Error in the system was less than 0.5% in position and less than 1.5° in angle [27]. Following calibration, the animal was placed on the treadmill. Kinematic data was recorded at treadmill speeds starting at 11 m/min, increasing to 21 m/min in 2 m/min increments. The speed was increased each time the animals completed at least 5 continuous stable step cycles (in the middle of the treadmill at a constant speed). The entire process took approximately 5–10 min per animal. If an animal was unable to complete all speeds on the first day, the task was repeated the next day. No animal took more than 2 days to complete all speeds.

Video was imported into Vicon Peak-Motus® software for analysis. The calibration object was digitized for odd and even fields to account for the de-interlacing of the frames done in Peak-Motus. A segment containing 5 stable step-cycles at 21 m/min was identified and imported into Peak-Motus. Video was manually synchronized using timestamps on the video. All reflective markers were digitized and the data was processed into joint angle versus time and marker 3D position data versus time. Gait cycles, swing, and stance phases were identified by marking lift-off (the first frame where the toe was not contacting the treadmill) and touchdown (the first frame where the toe contacted the treadmill) events for each limb. Joint angles and hindlimb coordination were assessed for changes with time. Data was scaled to the hindlimb cycle (with 200 points per cycle) and averaged across all cycles per animal. Specific joint angle values were also collected for swing and stance maxima and minima, the highest and lowest (respectively) values for joint angles during those gait cycle phases. Subcycle values are useful as neural control varies between stance and swing for reasons including loading and other afferent signals. Data from two of the animals (one sham and one iSCI) proved to be incompatible with the kinematic analysis software (most likely due to footfall event marking) and were excluded from most analyses. Unless specifically mentioned, n=5 animals per group were used.

### Coordination

In addition to displaying each joint independently, angle-angle plots were created to display information about coordination. Intralimb coordination was displayed as hip vs. knee, hip vs. ankle, knee vs. ankle, and shoulder vs. elbow. Interlimb coordination was displayed for each joint, left vs. right. These plots can be used for qualitative assessment of a number of features of locomotion [27,34]. A vertical or horizontal line represents movement in one joint while the other is held constant. A continuously changing phase relationship between the two joints is indicated by diagonal segments, with negative slopes indicating an out-of-phase relationship and positive slopes indicating an in-phase relationship. Finally, in the interlimb coordination plots only, symmetry around the y=x axis can be used to assess symmetry in the joint kinematics.

For quantitative analysis, phase delay was calculated for a number of intra- and interlimb combinations. Intralimb coordination was calculated for Hip-Knee and Hip-Ankle. Interlimb coordination was calculated for forelimb-forelimb, hindlimb-hindlimb, and forelimb-hindlimb. Following methods used previously [27,35], the relative phase of movement of one joint or limb was assessed with respect to its pair. For interlimb coordination, this involved comparing touchdown events between the respective limbs. For intralimb coordination, the point of maximum flexion for each joint during the swing phase was used. Each time point (footfall or maximum flexion) for the first half of the pair was denoted as *τa*_*i*_*, i = 0, 1, 2, . . . N* and the second of each pair as *τb*_*i*_*, i = 0, 1, 2, . . . N*, where each *τ* is the time point of that event, and *N* is the number of cycles. The phase of the second with respect to the first for step cycle *i* is then calculated using Equation 1 [36].

(1) $$\Phi \left(\mathrm{\tau a}b_{i}\right)=\frac{\left(\tau a_{i}-\tau b_{i}\right)}{\left(\tau a_{i+1}-\tau a_{i}\right)},\tau b_{i}<\tau a_{i}<\tau b_{i+1}$$

Multiple cycles are then averaged together. While left-right phase gives us some information about symmetry (the closer it is to 0.5 the more symmetric the gait is), a second symmetry measure was also used. In the following method, symmetry was assessed by using right side to predict the left side at each point in time. This measure used kinematics which were normalized to the gait cycle. Assuming the joint angle movement is symmetric (as it should be in normal animals), the right side joint angles can be used to predict the left side joint angles with a phase lag of half of the cycle. The difference between the point and its prediction (the symmetry error) can be calculated with Equation 2 [35].

(2) $$\mathrm{SY}M_{E}=\sqrt{\frac{{\sum_{i=1}^{n}\left(\frac{\theta R_{i}-\theta L_{i+\mathrm{hp}}}{\sqrt{2}}\right)}^{2}}{n}}$$

*θR*_*i*_ is the angle of the right side joint at point *i, θL(*_*i+hp*_*)* is the angle of the left side joint at the point one half the cycle period ahead of the right side, and *n* is the number of points in the cycle.

### Complexity measures

**Figure 1:**
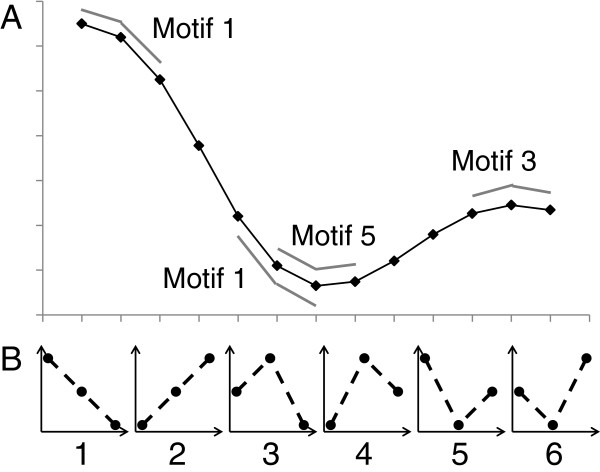
**Permutation entropy method.** Permutation entropy (PE) was calculated for both unaveraged hindlimb trajectories and cycle averaged trajectories for each joint angle. Permutation entropy quantifies the probability that a signal will remain similar from one segment to the next. Changes in the direction of the signal (positive to negative slope, for instance), indicate increases in complexity, while a steadily decreasing slope would indicate less complexity. A unitless number 0–1 describes the results, with higher values having higher complexity. **A**: The angle trajectories are segmented into 3 point motifs. **B**: The motifs are then classified in to one of 6 possible categories (1–6). PE is then calculated using the standard Shannon uncertainty formula.

Permutation entropy (PE) [37] was calculated for both unaveraged (raw) hindlimb trajectories and cycle-averaged trajectories for each joint angle. Entropy can be defined as “the average quantity of information obtained by observing a random variable” [38]. Permutation entropy quantifies the probability that a signal will remain similar from one time segment to the next. Changes in the direction of the signal (positive to negative or negative to positive slope) indicate increases in complexity, while a constant slope (continuously decreasing or continuously increasing signal) would indicate less complexity [39]. Thus, a signal with multiple phases per cycle would have higher complexity than one with only one phase per cycle. Permutation entropy ranges from 0 to 1, with higher values indicating higher complexity. As PE becomes closer to zero, the quantity of information decreases and fewer control signals are required to produce the movement. The reduction in needed control signals is a reduction in complexity. Our method used the MATLAB code published by Olofsen [40], based on the work of Cao [41] who used the measure to characterize complexity of electroencephalograms (EEG). First, angle trajectories were segmented into 3-point motifs. The motifs were then classified into one of 6 possible categories (Figure 1). The number of motif’s belonging to each category was counted to obtain the probability (*р*_*i*_) of each numbered (*i* = 1–6) motif occurring. PE was calculated using the standard Shannon uncertainty formula (Equation 3) [40].

(3) $$\mathrm{PE}\;=\;-\frac{\sum {}_{i}\left[p_{i}\;\times \;\ln\;\left(p_{i}\right)\right]}{\ln\;\left(\mathrm{number}\;\mathrm{of}\;\mathrm{motifs}\right)}$$

As mentioned, PE was calculated for both averaged trajectories (a compilation trajectory of both left and right sides for each joint) and raw, unaveraged trajectories for each joint separately. Unlike Olofsen, we only used Tau=1 (three point motifs), not a combination with Tau=2 (six point motifs) as there are not multiple wave frequencies in gait as there are in EEG.

In the context of time series data, principal component analysis (PCA) is used to assess the similarity of waveforms with one another. It is used to reduce the dimensionality of data or to determine the highest sources of variation within the data [42]. PCA was used to determine the loss in complexity in overall hindlimb gait following injury. If more variance in the gait data is accounted for by fewer principal components, then this may indicate that gait complexity has reduced, and the joints are moving more in synchronization with one another. When joints move in synchronization, the independence of the neural signals is reduced, thus fewer control signals are required to produce the movement. The reduction in needed control signals is a reduction in complexity. Thus, as the value for the first principal component (PC1, the percentage of variance account for by that component) increases, the complexity of the system decreases. As PC1 increases, less variance is available for remaining components. In the case of hindlimb angle trajectories, as PC1 approaches 100%, the three joints of the hindlimb become in phase with one another such that all three joints are flexing and extending together as opposed to each joint being controlled independently. This would be a much simpler pattern than is seen in normal subjects. Analysis was performed using the PRINCOMP function in MATLAB on the set of three hindlimb angle trajectories for both hindlimbs for each animal then averaged across groups (sham and iSCI). When using the three hindlimb joint trajectories instead of multiple subjects, the maximum number of the principal components is 3, so data reduction is limited. Instead of counting the number of principal components required to reach a specified variance, amount of variance accounted for by the first principal component was assessed. The method used was similar to those performed on a single joint angle across subjects [42-44], but the three hindlimb joint angles were compared as opposed to multiple subjects. The different time points represent the different “variables” in a standard PCA analysis. PCA was performed on each hindlimb of each animal and the proportion of variance accounted for by each of the three principal components was obtained.

### Muscle properties

Following 3D kinematics, rats were euthanized under heavy anesthesia (40 mg/kg sodium pentobarbital plus supplementary 1-5% isoflourane) and the hindlimb was separated from the remaining tissue. Hindlimb muscles were carefully dissected to lactated ringer’s solution. Muscles included gastrocnemius (medial and lateral heads), soleus, tibialis anterior, biceps femoris (knee and hip portions), semitendinosis, vastus (lateral and medial heads), and rectus femoris. All dissected muscles were weighed after light dabbing to remove surface liquid. Some (38 muscles from 5 animals, 3 sham, 2 contusion) muscles had their volume measured by displacement in a graduated cylinder; volume measurements were used only to calculate muscle density. Muscles were split along the belly and fiber pennation angle was measured using a goniometer as well as fiber length measured using a digital caliper under no significant tension. Individual fibers or fiber bundles were dissected from the belly of the muscle and placed on a glass slide with a small hole in the center under minimal tension. An 8 mW helium-neon (red) laser was beamed through the fiber and the first octave locations of the diffraction patterns were measured. Diffraction was converted to sarcomere length using Equation 4 [45].

(4) dsinθ=nλ

where d is the sarcomere length, θ is the angle of diffraction, n is the diffraction order, and λ is the laser wavelength.

### Statistical analysis

Differences in parameters between sham and iSCI groups were analyzed using a Wilcoxon-Mann–Whitney non-parametric test with p = 0.05 due to the small sample size. PE and PCA measures were conducted using standard t-tests, because in cases where both hindlimbs were measured independently, left and right limb values were considered repeated measures for data analysis. Variability has been reported as standard error (SEM) for data in which multiple cycles have been averaged together and standard deviations (SD) in all other cases. All statistical analyses were run in SAS 9.2 (SAS institute Inc, Cary, NC).

## Results

### Open field locomotion indicates mild-moderate injury

**Figure 2:**
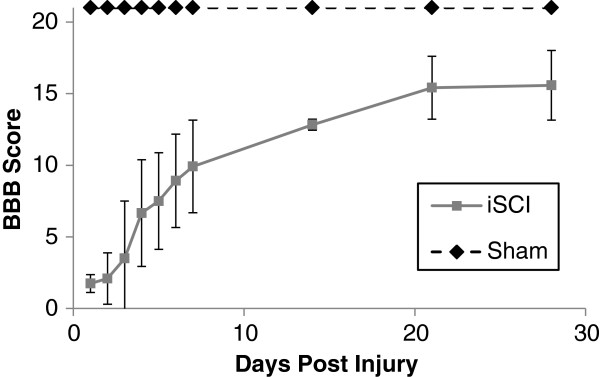
**Basso-Beattie-Bresnahan (BBB) locomotor scores for 4 weeks post injury.** Sham injured rodents had a score of 21 indicating no injury. The BBB score for the iSCI group increased from 2, at 1 day post injury and plateaued at approximately 15 indicating recovery of hindlimb weight support and forelimb-hindlimb coordination. Data are mean ± SD.

Sham injured animals showed normal locomotion on the BBB scale following injury. As seen in Figure 2, the initial deficits seen in the iSCI animals recovered to and plateaued at a BBB score of appropriately 15 (indicating hindlimb weight support with consistent forelimb-hindlimb coordination) by 2 weeks. Note the early recovery of hindlimb weight support (BBB 8–13).

### Passive muscle parameters indicate a lack of muscle atrophy

Table 1 summarizes all measured descriptive muscle parameter values. Muscle masses showed no difference between sham and iSCI groups. As expected, pennation angle, fiber length and sarcomere length also showed no difference. Pennation angle tended to be greater in the distal muscles than in the proximal muscles, with non-pennate muscles seen in the knee flexors and hip extensor. Average muscle density was 0.97 ± 0.11 g/ml for sham and 1.00 ± 0.14 g/ml for iSCI groups. Average animal masses were: 284 ± 10 g pre-injury sham, 283 ± 4 g pre-injury iSCI, 305 ± 15 g 4 weeks post-injury sham and 318 ± 24 g 4 weeks post-injury iSCI. All variability reported as SD.

**Table 1:** Hindlimb muscle properties in sham and iSCI rats

	**Muscle weight (g)**		**Pennation angle (deg)**		**Fiber length (mm)**		**Sarcomere length (mm)**	
	**Sham**	**iSCI**	**Sham**	**iSCI**	**Sham**	**iSCI**	**Sham**	**iSCI**
GM	0.80 ± 0.10	0.82 ± 0.10	29.7 ± 9.0	23.3 ± 5.6	10.63 ± 4.53	7.77 ± 0.62	1.99 ± 0.15	2.12 ± 0.23
GL	1.24 ± 0.20	1.18 ± 0.11	31.0 ± 7.4	27.3 ± 7.6	10.92 ± 6.57	9.93 ± 2.36	2.03 ± 0.07	2.10 ± 0.19
SOL	0.18 ± 0.03	0.18 ± 0.02	24.3 ± 11.2	21.0 ± 1.4	9.81 ± 3.04	8.69 ± 3.70	2.23 ± 0.11	2.14 ± 0.23
TA	0.69 ± 0.06	0.66 ± 0.03	32.3 ± 11.6	26.3 ± 9.0	13.32 ± 3.16	11.72 ± 3.98	2.24 ± 0.32	2.07 ± 0.25
VL	1.13 ± 0.09	1.22 ± 0.13	28.9 ± 4.6	27.3 ± 6.6	12.78 ± 1.08	13.67 ± 2.21	2.09 ± 0.06	2.09 ± 0.39
VM	1.60 ± 0.08	1.51 ± 0.17	21.8 ± 17.9	22.5 ± 3.5	11.96 ± 4.21	14.76 ± 2.63	2.24 ± 0.40	2.18 ± 0.06
RF	0.88 ± 0.15	0.86 ± 0.07	27.3 ± 2.1	34.2 ± 4.3	8.25 ± 1.59	8.19 ± 1.87	2.02 ± 0.19	1.95 ± 0.15
ST	1.28 ± 0.23	1.22 ± 0.27	0.0 ± 0.0	0.0 ± 0.0	29.96 ± 3.49	26.18 ± 5.15	2.12 ± 0.18	2.05 ± 0.14
BFk	2.09 ± 0.33	2.12 ± 0.27	0.0 ± 0.0	0.0 ± 0.0	26.92 ± 1.70	22.45 ± 3.69	1.99 ± 0.17	2.03 ± 0.06
BFh	1.02 ± 0.24	0.90 ± 0.06	0.0 ± 0.0	0.0 ± 0.0	25.87 ± 9.85	22.70 ± 5.77	2.01 ± 0.09	1.94 ± 0.17

Muscle weight, pennation angle, fiber length and sarcomere length were obtained for Gastrocnemius Medialis (*GM*), Gastrocnemius Lateralis (*GL*), Soleus (*SOL*), Tibialis Anterior (*TA*), Biceps Femoris-hip extensor (*BFh*), Biceps Femoris-knee flexor (*BFk*), Semitendinosis (*ST*), Vastus Lateralis (*VL*), Vastus Medialis (*VM*), and Rectus Femoris (*RF*). Statistically significant differences were not observed for any measures between groups, suggesting absence of muscle atrophy. Standard deviation was also similar between groups for all measures. Data are Mean ± SD, n = 5 per group.

### 3D locomotor kinematics indicate decrease in complexity

**Figure 3:**
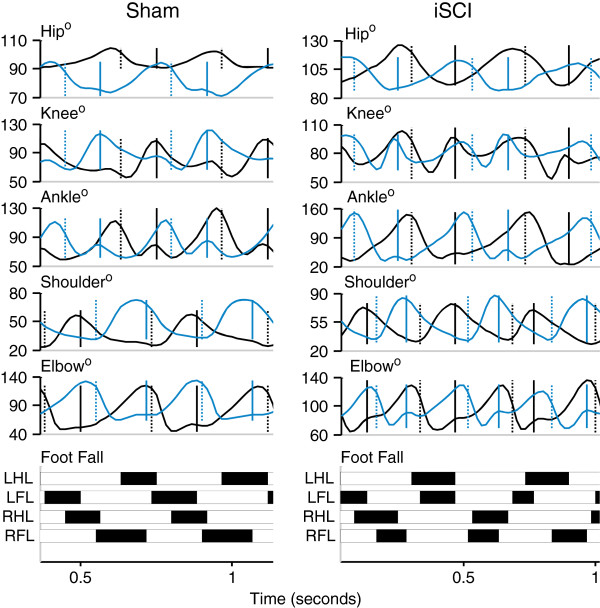
**Example locomotor kinematics during treadmill walking.** Joint angle kinematics and footfall measures during treadmill walking from one sham and one iSCI animal (T9 spinal level), 4 weeks post injury. The black traces are for the left limb and the blue traces are for the right limb. The solid vertical lines are touchdown and the dotted vertical lines are lift-off. For footfall, the black sections represent swing and the white sections stance. LHL, left hindlimb; LFL, left forelimb; RHL, right hindlimb; and RFL, right forelimb. Note the decrease in the magnitude of the second local maxima of the ankle trajectory in the contusion animal as well as the inconsistent step pattern.

**Figure 4:**
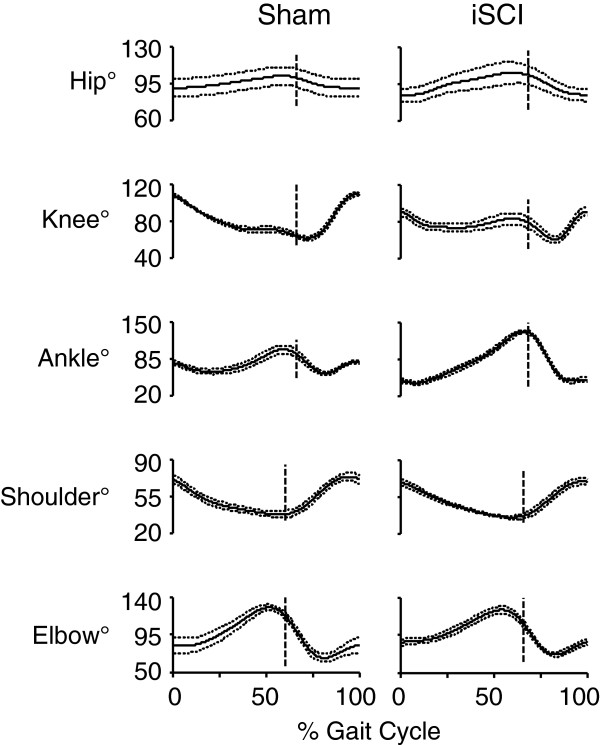
**Averaged limb joint angles averaged across all cycles for all animals.** Limb joint angle trajectories for each measured joint averaged across all cycles of all animals. Range of motion in the ankle was greater in the iSCI group, particularly due to overextension toward the end of the stance phase. Additionally, the angle trajectory of the iSCI animals shows one instead of two local maxima as seen in the sham group. The second local maximum is a result of pre-activation of the gastrocnemius in preparation for touchdown. As expected, forelimb trajectories are mostly unchanged following injury. Data are mean ± SEM degrees, n = 5 per group. Vertical line indicates lift-off.

**Figure 5:**
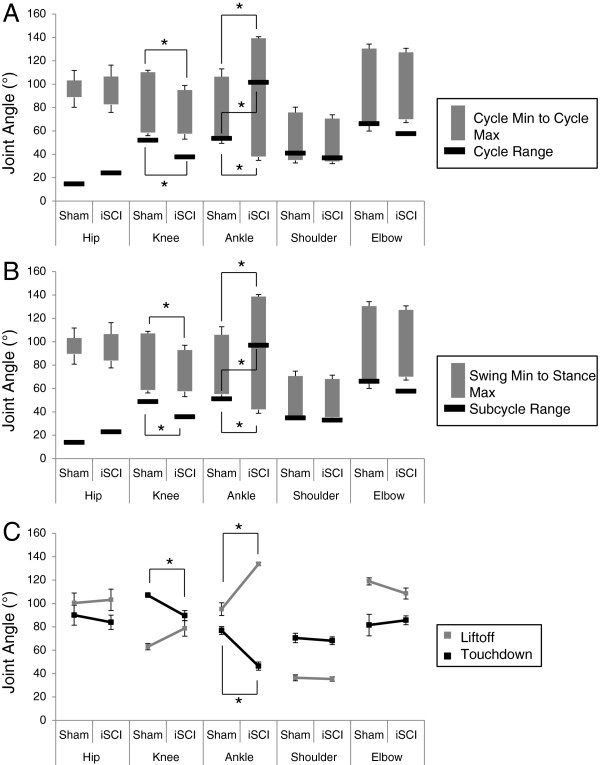
**Joint angle measures at specific points in the gait cycle. A**: Joint angle values for cycle minimum, cycle maximum and cycle range for all measured joints for both sham injury and incomplete spinal cord injury (iSCI) groups. Large increases were noted in all measures at the ankle, particularly at cycle max (overextension) and in cycle range when comparing iSCI to sham animals. The range of motion and cycle max for the knee were decreased in iSCI animals when compared with sham. Mean ± SEM degrees for 5 rats per group. (*) indicates p<0.05. **B**: Joint angle values for swing phase minimum, stance phase maximum and subcycle range for all measured joints for both sham injury and incomplete spinal cord injury (iSCI) groups. Large changes were noted in all measures at the ankle, particularly in stance max (overextension) and subcycle range as well as two measures at the knee. No significant changes were noted at the hip. Mean ± SEM degrees for 5 rats per group. (*) indicates p<0.05. **C**: Joint angle values for lift-off and touchdown for all measured joints for both sham injury and incomplete spinal cord injury (iSCI) groups. In the sham animals, knee angles values at lift-off and touchdown are very different from one another and ankle angle values are very similar to one another. For iSCI animals, the opposite is true. Knee values become similar to one another and ankle values become vastly different. Mean ± SEM degrees for 5 rats per group. (*) indicates p<0.05.

Large differences in coordination were seen between sham and iSCI animals. Individual traces from single animals from each group highlight inconsistency in footfall patterns and decrease in magnitude of the second local maxima in the ankle joint angle trajectory in the iSCI animals (Figure 3). As seen in Figure 4 (averaged across the groups), at each hindlimb joint, there was a noted change in the angle trajectories. The largest difference was seen in the ankle. During the ankle joint excursions, only one local maximum was seen as opposed to two pre-injury (one during stance and one during swing). The hip trajectory, was not qualitatively different following injury. At the ankle, each of the event joint angle values was different between the sham and iSCI groups for the entire gait cycle (Figure 5A). In particular, ankle range of motion and lift-off value for the joint angle were much higher in the iSCI group than the sham group. The knee had a lower range of motion and lower cycle maximum following injury. When the range was restricted to swing maximum to stance minimum, the results remain the same (Figure 5B). In the sham animals, knee angle values at lift-off and touchdown were very different from one another and ankle angle values were very similar to one another (Figure 5C). For iSCI animals, the opposite was true. Overall the largest number of changes was seen at the ankle, followed by the knee, with no noted gait changes at the hip. Due to the large number of multiple comparisons made in Figure 5, the results of any single test need to be interpreted cautiously, but the overall pattern of results (ankle versus knee versus hip) paints a compelling picture of the joint specific nature of the results and suggests candidates for future testing.

**Figure 6:**
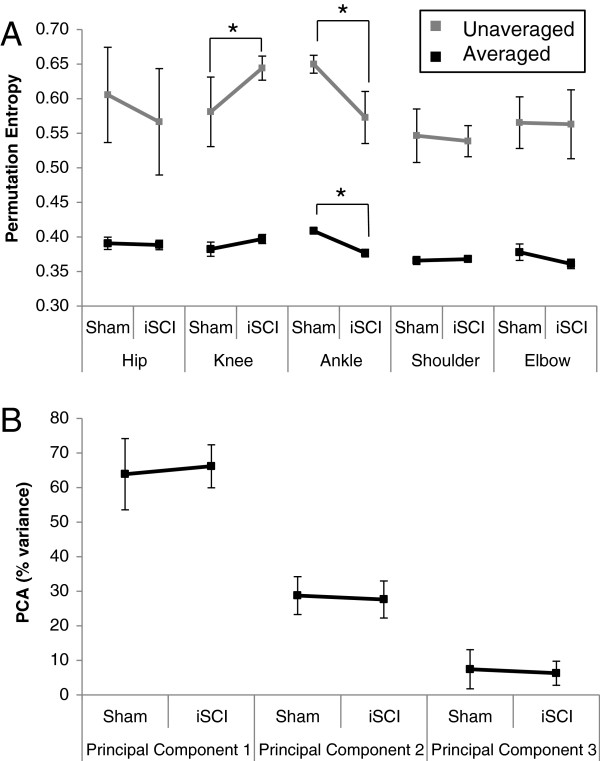
**Locomotor complexity. A**: Permutation entropy for unaveraged limb trajectories (gray, 5–12 cycles per animal, n=6 per group; Mean ± SD) and averaged trajectories (black, n=5 per group; Mean ± SEM). Lower values indicate lower complexity. As averaging removes information, the averaged data have lower complexity. A decrease in ankle complexity was noted in both the averaged and unaveraged data. An increase in complexity was note in the knee but only for the averaged data. This may indicate that the changes in the knee were in step-to-step variation only. (*) indicates p<0.05. **B**: Percent of variance accounted for by all three principal components from PCA analysis. Larger PC1 indicates lower complexity. No significant differences were noted. n = 5 per group.

Permutation entropy of the unaveraged limb joint angle trajectories indicated a decrease in complexity of movement at the ankle with a corresponding increase in complexity at the knee (Figure 6A). The decrease in ankle movement complexity was likely due to the loss of the second local maximum in the trajectory (the ankle goes from biphasic to monophasic). The increase in knee movement complexity may mean the opposite. No other significant differences were found. Permutation entropy of averaged joint angles continued to show the decrease in movement complexity at the ankle, but with no commensurate increase at the knee. This may indicate that the change to the complexity of movement at the knee may reflect step-to-step variations while the changes to the movement complexity at the ankle reflect within cycle changes. The un-averaged PE measure may be subject to error due to the differing cycle period between animals; however, the use of the same treadmill speed for each animal should minimize this error.

**Figure 7:**
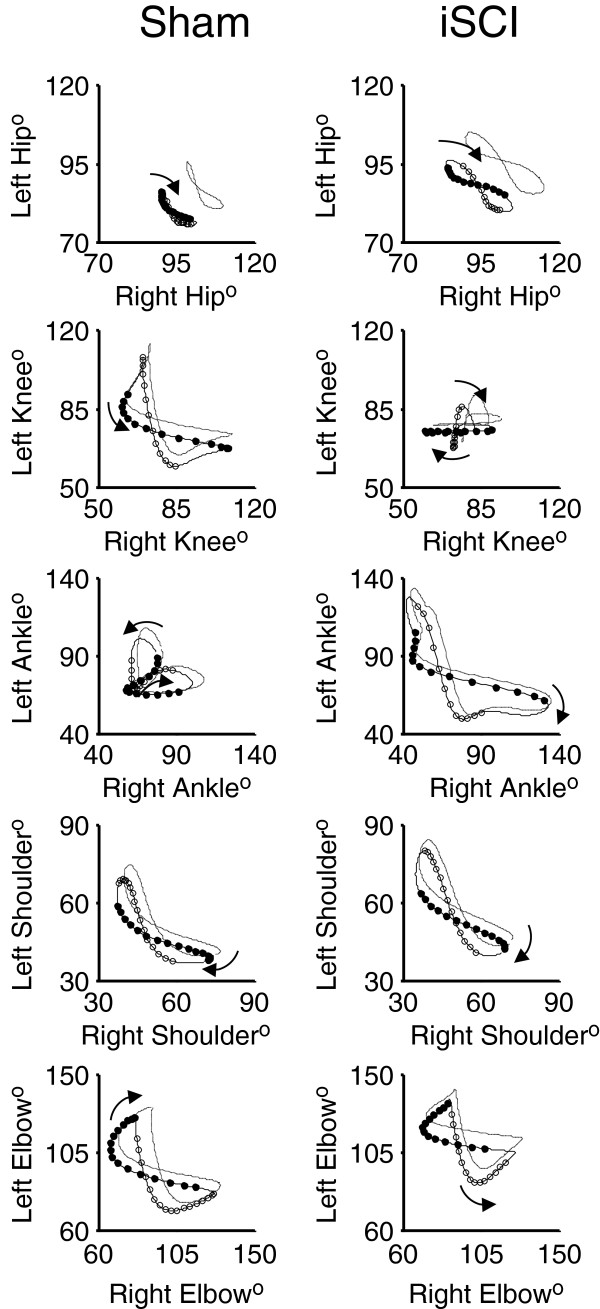
**Interlimb coordination during locomotion.** Left-right coordination plots for sham and incomplete spinal cord injury (iSCI) groups. Following injury, symmetry is maintained about the diagonal. Ankle-Ankle plots are simplified due to weakened control signals to the distal joint and the loss of the biphasic nature of the joint angle kinematics. Knee-Knee plots show a cruciform pattern where one side is moving only when the contralateral side is not. Forelimb coordination is mostly unaffected by the injury. Lines are mean (solid) plus SEM (dashed). Circles mark a time spacing of 8.33 ms with solid indicating stance and open indicating swing. n = 5 per group.

**Figure 8:**
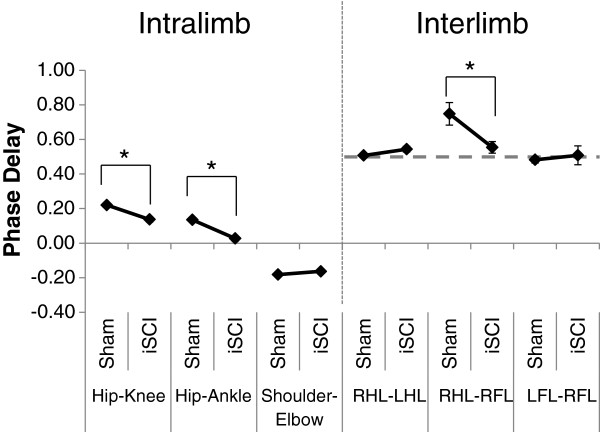
**Phase delays for inter- and intralimb coordination.** Phase delays are represented by proportion (0–1) of gait cycle out of phase. Phase values are calculated as lead (positive value) or lag (negative value) of the maximum flexion or touchdown for the second item with respect to the first item. Hip-Knee, hip to knee maximum flexion; Hip-Ankle, hip to ankle maximum flexion; Shoulder-Elbow, shoulder to elbow maximum flexion; RHL_LHL, right hindlimb to left hindlimb touchdown; RHL_RFL, right hindlimb to right forelimb touchdown; and LFL_RFL, left forelimb to right forelimb touchdown. Mean ± SEM for 5 rats per group. (*) indicates p<0.05 when compared with sham. Hindlimb-Forelimb (HL-FL) becomes completely out of phase. Decrease in Hip-Knee and Hip-Ankle delay: both become more in-phase with one another. No changes in forelimb (Shoulder-Elbow) or left-right phase delays (forelimb or hindlimb). Dashed gray line represents a phase relationship of 0.5 (completely out of phase).

Interlimb coordination waveforms (L-R joint comparisons) emphasized the changes due to injury with significant differences seen in the knee and ankle coordination following injury (Figure 7). Ankle-ankle plots were simplified due to lack of the second local maxima in the individual angle trajectories. Knee-knee plots showed a cruciform pattern, where one side is moving only when the contralateral side is not. Forelimb coordination was mostly unaffected by the injury. These observations of joint angle trajectory profiles obtained four weeks post injury were similar to those reported for animals with similar levels of contusion two weeks post injury but receiving neuromuscular electrical stimulation therapy [35]. As is visually clear from the interlimb left-right coordination plots and confirmed by the symmetry error measurements, there was no decrease in symmetry following iSCI for any of the hindlimb joints. In fact, there was more symmetry at the knee following injury (sham: 10.7° ± 1.1° SEM, iSCI: 7.7° ± 0.3° SEM, p=0.03), though the difference is small. Left-right hindlimb and left-right forelimb pairs showed no difference in phase delay between groups (Figure 8). This is somewhat expected as these animals were walking quite competently. Forelimb-hindlimb coordination was significantly altered, however. The phase delay was shifted approximately 50% (sham: 0.75 ± 0.065 SEM, contusion: 0.55 ± 0.034 SEM, p<0.05). This indicates that the gait shifted from a traditional walk pattern (each limb ~25% cycle delayed from the previous one) to something like a trot (where forelimb-contralateral hindlimb pairs are alternating 50% out of phase with one another) [46].

**Figure 9:**
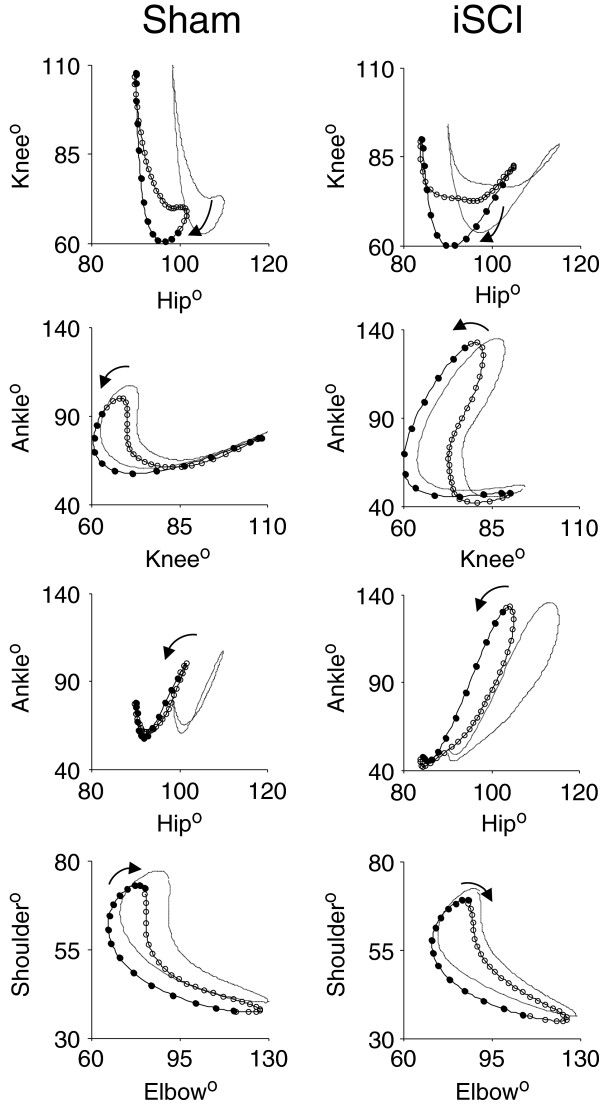
**Intralimb coordination during locomotion.** Intralimb joint-joint coordination plots for sham and incomplete spinal cord injury (iSCI) groups. Forelimb intralimb coordination is expectedly unaffected by the injury. Hip-Ankle coordination simplifies significantly with the two joints extending and flexing in unison. Ankle-Knee plots show a loss of the second co-extension phase with the ankle pre-extended before the knee begins extension. Lines are mean (solid) plus SEM (dashed). Circles mark a time spacing of 8.33 ms with solid indicating stance and open indicating swing. n = 5 per group.

Intralimb coordination plots were also simplified, particularly when looking at the Hip-Ankle (Figure 9). Plots that lie close to the y=x or y=−x diagonal reflect joint motions that are occurring in sync with one another, as opposed to the more temporally complex pattern of joint motion seen in normal gait . Hip-Ankle coordination simplified significantly, with the two joints extending and flexing in unison for much of the gait cycle. Ankle-Knee plots showed a loss of the second co-extension phase with the ankle pre-extended before the knee begins extension. Sham and iSCI animals showed significantly different Hip-Knee and Hip-Ankle phase delays as well (Figure 8), confirming the qualitative assessment in the hindlimb intralimb coordination figures. Forelimb intralimb coordination was unaffected by the injury, as expected. These data are also similar to those reported in rats two weeks post injury that received 5 days of electrical stimulation therapy [35].

PCA analysis quantified the complexity of movement for the entire hindlimb by assessing the three hindlimb angle trajectories for each individual animal’s kinematics. Analysis was performed on the unaveraged joint kinematics of each leg of each animal and averaged together for each group (sham and iSCI). Each component’s contribution to the overall variance of the hindlimb kinematics was compared between groups in Figure 6B. While no significant results are noted, there is a trend (p=0.1083) toward an increase in PC1, which would indicate a decrease in complexity in the iSCI group. The lack of significance may be due to the small sample size and minimal impact to gait by the 4th week following a mild-moderate injury. The first components of the PCA analysis most likely account for the gait cycle and the coordination between the flexors within the limb (and likewise the extensors) while the second and third components represent the intricacies of the joint trajectories. As the first component accounts for more and more of the variance, the three joints of the limb become more in phase with one another such that they could be controlled by a single neural signal.

## Discussion

In this study, data were collected from a set of mild to moderately injured rats in the chronic phase of recovery (4 weeks post-injury) where muscle atrophy was negligible, but significant locomotor impairments were still seen during walking. These impairments resulted in changes in kinematic complexity due to changes in neural control. The ankle specifically was particularly sensitive to loss of supraspinal control. No study to date has shown a decrease in kinematic complexity following incomplete spinal cord injury in rat, particularly when overall gait recovery was as complete as shown here. We also showed that changes in kinematic complexity were joint specific, indicating that different joints are under differing control in locomotion. Finally we collected muscle parameters from iSCI rats showing no indication of atrophy.

### Lack of change in muscle parameters

Muscle parameters were collected to determine presence or lack of atrophy for iSCI rats four weeks post injury. Gregory et al. [47] looked at muscle disuse following complete transection injury in order to assess interspecies differences between rats and humans, but only assessed fiber type changes, not atrophy. Liu et al. [48] compared cross sectional area of hindlimb muscles for 18 weeks following a contusion injury (BBB: 11.1). They showed muscle atrophy in all muscles for the first 3 weeks following injury, with a gradual return to pre-injury values starting at week four, with all muscles recovering by 12 weeks. As their injury level was slightly higher than in this study and a different strain was used (Sprague–Dawley), the minor difference in results is consistent with our findings.

In a study of contusion injured rats, Hutchinson et al. [4] measured changes in muscle masses following injury. They used a higher level of injury (approximately 4–5 points lower in the BBB) and observed atrophy (~19%) in the gastrocnemius even 10 weeks post injury. In the current study, higher BBB scores were observed, indicating a lower level of injury. This led to weight bearing occurring a few days earlier, thus ameliorating the effects of the injury on muscle atrophy [49]. Also of note, the rats in the current study gained weight following injury, while the rats in Hutchinson et al.’s study lost weight, possibly leading to an increase in atrophy rate. When compared to a weight-matched set of sham controls, the muscle masses for rats in the current study are approximately 1% (TA) to 23% (soleus) greater than those reported by Hutchinson. This could be due to differences in strain.

### Changes in locomotor coordination in absence of atrophy

While similar data reduction techniques applied to a large number of EMG signals following stroke have been successful [16], PCA has not been applied to rat kinematic data in this fashion before. The analysis technique could be an important tool to assess gait with higher levels of injury or larger sample sizes. The lack of significant results could be complicated by the fact that the 3 joint trajectories are from a single animal so the results may appear different than those used in other types of PCA analysis of joint kinematics where each joint (hip, etc.) is assessed independent from one another [42].

In the intra-limb coordination plots (with the exception of the Knee-Hip plot), the waveforms appear to collapse to a diagonal in the injured animals, implying that the joint motions for injured animals become coordinated around just two control signals (flexion and extension) as seen in stroke [16]. This is most noticeable in the Ankle-Hip plot. Increased joint synchronicity in injured animals is also supported by the hindlimb phase delay data, which showed a decrease in the Hip-Ankle phase delay in the iSCI animals; the peaks of the two angle trajectories came closer to one another in time. Ankle and knee joints, however, became more out of phase with one another following injury. Thus the decrease in phase delay between the hip and ankle (which would indicate a decrease in complexity in the PCA) may have been counteracted by the increase in phase delay between the ankle and knee (which would indicate an increase in complexity in the PCA).

As iSCI animals in this study did lose the second ankle angle peak but did not have muscle atrophy, this loss of kinematic complexity is most likely due to loss of supraspinal control (a delay in gastrocnemius EMG burst initiation [27]) and not loss of muscle strength. Finally, as indicated in Figure 4, the overextension in the ankle (likely due to the loss of supraspinal control [50]) and lower range of motion in the knee tended to counteract each other, indicating that the remaining impaired control signals to the hindlimb can be used to establish correct foot position [51].

The cruciform pattern seen in knee-knee intralimb coordination (Figure 8) is not unique to this study. In Jung et al. [52], rats were given the same level of injury as reported in this study. One week following the injury they were given 5 days of patterned electrical stimulation to the hip muscles in order to produce a locomotor pattern at the hip. 3D kinematics were then collected 14 days post injury (dpi). In both the current study and Jung, BBB scores indicated that the animals were nearly recovered; however, the animals permanently altered their gait as indicated by kinematics. The post-therapy animals developed a cruciform pattern in the Knee-Knee angle plots [35] (14 dpi, 7 days post therapy) similar to that observed in the iSCI animals in the current study at 28 dpi, suggesting that the stimulation therapy accelerated recovery following injury. While this pattern was different than that seen in normal animals, the animals were able to reach the targeted treadmill speeds with ease. Along with this similarity was one in forelimb-hindlimb coordination. In both Jung and the current study, forelimb-hindlimb coordination shifted from approximately 50% out-of-phase to 100% out-of-phase. As this gait is stable and effective, it is a successful endpoint for locomotor recovery despite not matching pre-injury patterns.

### Joint-specific changes in locomotor complexity

While PCA analysis did not prove a decrease in complexity in the whole limb kinematics, PE analysis of individual joint kinematics showed joint-specific changes in complexity. PE also showed itself to be a quantitative measure of changes in joint angle trajectory. Changes in PE at the ankle were accompanied by changes in ankle angles. While analysis of control of locomotion tends to focus on the joints as similar structures (i.e., receiving similar input from sensors, supraspinal sources, and the central pattern generator (CPG) [53]), the results of this study emphasize the importance of also considering specific roles for individual joints [54-56]. In general, all joints do receive similar input from all sources of locomotor control. A tonic drive from the brain initiates gait and provides feed-forward adaptations to perturbations [57,58]. The CPG and pattern shapers produce the basic profile for gait [59]. Sensory information from muscles spindles, Golgi tendon organs, and cutaneous receptors modify the gait pattern [53,60]. A number of specific aspects of gait are less general than this however, with each joint being affected differently by feed-forward supraspinal control or reflex sensory input during different phases of the gait cycle. Data from guinea fowl show a proximal to distal gradient in neuromechanical control; proximal muscles use more feed-forward control and the distal muscles less [55].

Ankle muscle amplitude is as much as 70% controlled by local sensors [61] which may be even greater when supraspinal control is decreased [62]. This may be due to a very high proprioceptive gain in the ankle muscles [63,64]. Due to the high reliance of the ankle on sensory input for motor control, the lack of significant muscle forces or cutaneous input during swing may contribute to the ankle flexor muscles (Tibialis Anterior) continued activation until the end of stance. This would lead to the lack of the normal end swing ankle extension that was noted in the iSCI animals in this study. Studies in humans indicate that the ankle has little to no feed-forward control following incomplete injury while the hip still maintains a large feed-forward component [54]. In the absence of planned foot placement, the ankle stays flexed for the duration of swing and loses its second minima/maxima. This hypothesis is further supported by the type of injury sustained. The contusion injury model used strikes the dorsal surface of the cord leading to significant damage to the dorsal tracts [33]. In the rat, the primary dorsal tracts are the dorsal corticospinal tract, the fasciculus gracilis (trunk and hindlimbs) and the fasciculus cuneatus (forelimbs) [65,66]. In rats, the corticospinal tract serves a very limited purpose, only directly controlling individual digit movements [67,68]. However, it also serves to modulate lumbar stretch reflexes [67]. As the ankle is primarily controlled by local reflexes, damage to the dorsal corticospinal tract would lead to significant changes in ankle control.

As with the initiation of foot swing, the ankle tends to act as a controller for the hip, with ankle-foot loads modulating hip torques [54,69]. The lack of feed-forward control at the ankle may serve a positive purpose. In guinea fowl, feed-forward planning for perturbations during overground locomotion has a more negative effect on gait than an unplanned perturbation [70].

One of the major functions of the knee is to couple the hip to the ankle. It contains many multiarticular muscles, coupling it with the proximal and distal joints. Knee extensors are synergistic with ankle extensors and inhibit ankle flexors [71]. The extensors in the hip are coupled to the knee via the stretch reflex [72]. Changes in the knee joint angle excursions are then likely due to the changes in control of the other joints [73]. Further investigations will thus be necessary to determine the actual control mechanism at play at each joint and the overall limb.

## Conclusions

There are three major conclusions in the paper. 1.) Locomotor changes (particularly decrease in locomotor “complexity”) following mild contusion injuries in rats happen in the absence of muscle changes allowing us to minimize the contributions of muscle changes in interpretation of the results. 2.) Changes in gait parameters are joint-specific, with more changes occurring the more distal the joint. 3.) Permutation entropy may be used to quantify differences in joint angle trajectories in a way that may be relevant to motor control (complexity and related quantity of neural control). This is the most evident when looking at the loss of the second local maxima in the ankle trajectory, but the measure can detect smaller changes as well.

In this study, treadmill walking was used to assess locomotor capabilities. The data showed that changes in kinematic complexity were joint-specific, indicating that different joints are under differing control in locomotion. Specifically, the ankle showed a decrease in complexity of movement, likely due to its unique role in locomotion. The ankle showed a decrease in kinematic complexity accompanied by changes in other ankle kinematic measures, supporting the use of the PE measure for gait analysis. Animals in this study showed a complete lack of changes in measured muscle parameters. This may indicate that weight bearing within 6 days following injury is sufficient to ameliorate the disuse-induced changes in the muscles. To our knowledge, no previous study has analyzed changes in kinematic complexity along with known levels of muscle atrophy.

Despite the injury, the animals do achieve stable and efficient gait, able to reach the desired 21 m/min speed used for data collection. Following the injury, communication between the hindlimbs and the brain is impaired and locomotor control shifts predominantly towards that of the local circuitry of the spinal cord and sensors. As such, a more primitive gait is unmasked. While rats may regain effective locomotion following injury, brain-spinal communication is never fully restored and thus the animal must adapt to use the local hindlimb circuitry for locomotion to become proficient in gait. This data suggests that evaluation of hindlimb EMG, specifically timing of bursts, may further demonstrate the loss of complexity in movement seen following injury, particularly at the ankle. This work may provide the basis for new strategies for rehabilitation interventions following spinal injury or modify existing ones by changing the rehabilitation parameters on a joint-specific basis, for instance, adding active ankle control to existing robotic interventions which currently use passive ankle control. Additionally, the study provides a novel set of muscle data that could be useful in the development of an empirically derived neuromusculoskeletal computational model for the rodent hindlimb.

## Competing interests

The authors declare that they have no competing interests.

## Authors’ contributions

BKH was responsible for data collection, data analysis, some experimental design, and the lead on manuscript writing and figure design. GY contributed to experimental design. JA contributed to experimental design and manuscript preparation. RJ contributed to experimental design and manuscript preparation and gave final approval of the version to be published. All authors read and approved the final manuscript.
